# 2-D Impulse Noise Suppression by Recursive Gaussian Maximum Likelihood Estimation

**DOI:** 10.1371/journal.pone.0096386

**Published:** 2014-05-16

**Authors:** Yang Chen, Jian Yang, Huazhong Shu, Luyao Shi, Jiasong Wu, Limin Luo, Jean-Louis Coatrieux, Christine Toumoulin

**Affiliations:** 1 Laboratory of Image Science and Technology, Southeast University, Nanjing, China; 2 The Key Laboratory of Computer Network and Information Integration (Southeast University), Ministry of Education, Beijing, China; 3 Centre de Recherche en Information Biomedicale Sino-Francais (LIA CRIBs), Rennes, France; 4 Research Center of Mixed Reality and Advanced Display, Beijing Institute of Technology, Beijing, China; 5 INSERM, U1099, Rennes, France; 6 Université de Rennes 1, LTSI, Rennes, France; Universidad Veracruzana, Mexico

## Abstract

An effective approach termed Recursive Gaussian Maximum Likelihood Estimation (RGMLE) is developed in this paper to suppress 2-D impulse noise. And two algorithms termed RGMLE-C and RGMLE-CS are derived by using spatially-adaptive variances, which are respectively estimated based on certainty and joint certainty & similarity information. To give reliable implementation of RGMLE-C and RGMLE-CS algorithms, a novel recursion stopping strategy is proposed by evaluating the estimation error of uncorrupted pixels. Numerical experiments on different noise densities show that the proposed two algorithms can lead to significantly better results than some typical median type filters. Efficient implementation is also realized via GPU (Graphic Processing Unit)-based parallelization techniques.

## Introduction

Median type filters are known as the effective methods to suppress impulse noise which often arises from sensor damage, malfunctioning or timing errors in signal acquisition [1]. Suppressing impulse noise routinely includes two stages - noise detection and noise removal [2]–[4]. The Standard Median Filter (SMF) is ineffective in cases with high noise densities, and the SMF processed images are often seriously degraded by detail loss and new introduced artifacts when the noise density is over 50% [5].

Several methods have been developed to overcome the drawback of SMF. Adaptive Median Filter (AMF) was proposed in [6] to improve the performance of median filter by only replacing corrupted pixel values by median values while leaving uncorrupted pixels unchanged. Additionally, the AMF recursively enlarges the filter window until one uncorrupted median is identified. The Decision Based Algorithm (DBA) proposed in [7] is similar to the AMF method except that DBA replaces the corrupted pixel value by a neighborhood pixel value when the median is tagged as a corrupted pixel. In [8], a method named Modified Decision Based Unsymmetric Trimmed Median Filter (MDBUTMF) was developed to improve DBA method by removing uncorrupted pixels in median calculation and setting the corrupted pixel value to mean of the selected window when all the window elements are corrupted. To achieve edge-preserving noise suppression, Chan and Nikolova put forward in [9] a two-phase algorithm which includes a SMF based noise detection and an edge-preserving term regularized optimization. In [10], a multi-scale adaptive median-based filtering was developed to suppress salt-and-pepper noise.

In suppressing impulse noise, median-type methods suffer from the image degradation of detail blurring and staircase artifacts. This problem might be more serious for high noise density cases [11], or the case combined with structure blurring [12]. The reason can be ascribed to the fact that only the median information from one single neighboring pixel is selected for median filtering and no structure continuity information is utilized. To overcome this, we propose in this paper an approach termed Recursive Gaussian Maximum Likelihood Estimation (RGMLE) for impulse noise removal. We focus this study on noise removal stage, so only salt-and-pepper type impulse noise with pre-known intensities is considered here. The proposed RGMLE approach solves the maximum likelihood estimation (MLE) of the corrupted points through recursive variance-weighted averaging. A robust strategy of controlling the recursion in RGMLE is also developed by analyzing the estimation error of the uncorrupted pixels. Under the framework of RGMLE, we derived two noise removal algorithms RGMLE-C and RGMLE-CS by respectively using certainty and joint certainty & similarity to estimate the variance. GPU (Graphic Processing Unit)-based technique is also applied to accelerate the proposed processing. Extensive experiments are performed to show that the RGMLE-C and RGMLE-CS algorithms can produce significantly improved results than the comparative median type filters. Among all the methods, the RGMLE-CS algorithm demonstrates the best restoration performance.

The structure of this paper is organized as follows: In section II, we explain the RGMLE approach together with the two derived algorithms RGMLE-C and RGMLE-CS. Experimental results are given and discussed in section III. Section IV concludes this paper with a brief description of its contributions and some open questions for future work.

## Reursive Gaussian Maximum Likelihood Estimation

**Figure 1:**
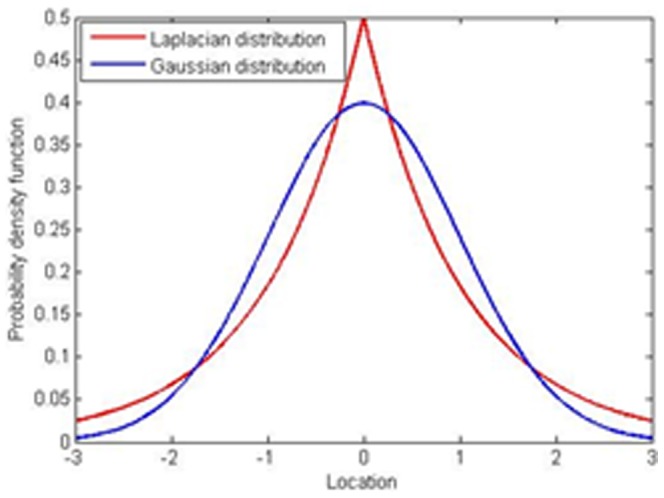
The probability density functions for Laplacian and Gaussian distributions.

Based on the theory of statistical estimation, the sample median corresponds to the maximum likelihood estimators of locations for independent and identically distributed (i.i.d.) observations obeying the Laplacian distributions with probability density function (pdf) shown in Fig.1 [13]–[14]. Let 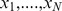 be *N* i.i.d. Laplacian distributed observations with unknown location parameter 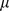 and the same variance 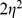, the MLE of 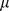 is denoted by 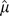, which maximizes the likelihood function:
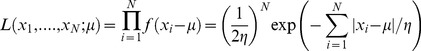
(1)This is equivalent to minimizing (2):
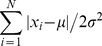
(2)Minimizing (2) with respect to 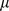 leads to the simple median 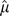 = 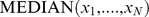 [13]. Here for an ordered list, the MEDIAN operator is defined as producing the middle value for odd number of values or the arithmetic mean of the middle two observations for even number of values. Assuming the Laplacian distribution of the observations in the 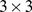 window, the 2D median filtering of the center pixel value 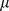 is in fact the maximizer of the likelihood function with respect to each location parameter 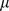 [15]–[16].

Likewise, with the Gaussian distribution shown in Fig. 1, we can build the likelihood function as follows[17]–[18]:
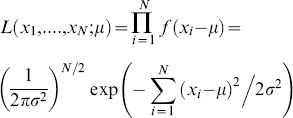
(3)The value 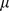 minimizing (3) leads to the average operator:
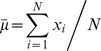
(4)Then we can easily obtain the variance 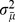 of 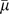:
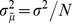
(5)Here, equation (5) shows that the variance estimation for Gaussian distribution decreases linearly with the available observation numbers.

However, in 2-D impulse noise suppression, assuming an identical variance for all samples in (3), the averaging filtering often leads to a non-discriminative averaging operation and might tend to produce oversmoothing in the restored images(we would illustrate this in the following section on experiment). Improved performance can be expected if a discriminative estimation of variance is applied based on the different reliabilities of different observations. Furthermore, considering the fact that the estimation error (or variance) decreases as the available uncorrupted observations increase, the information from corrected intensities should be exploited in a recursive way to refine the final restoration. In this work, we develop a Recursive Gaussian Maximum Likelihood Estimation (RGMLE) for impulse noise suppression. In this 2-D noise suppression case, with *y* the noisy image and 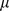 the target restored image, to estimate each corrupted pixel 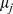 we can rewrite the likelihood function with variances 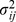 for each observation 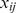 in the filter window 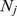:
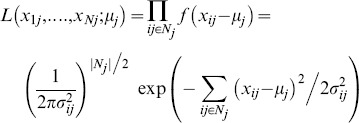
(6)Here 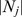 denotes the point number in filter window 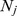. In (6), spatially-adaptive variance is used based on joint certainty and similarity information of observations, which is to be elaborated in detail below. Minimizing (6),with respect to 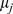 leads to a normalized variance-weighted average:
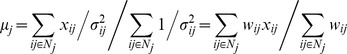
(7)Here, for each observation *i*, the weight 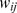 is a positive value calculated as the direct reciprocal of the variance 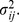


**Figure 2:**
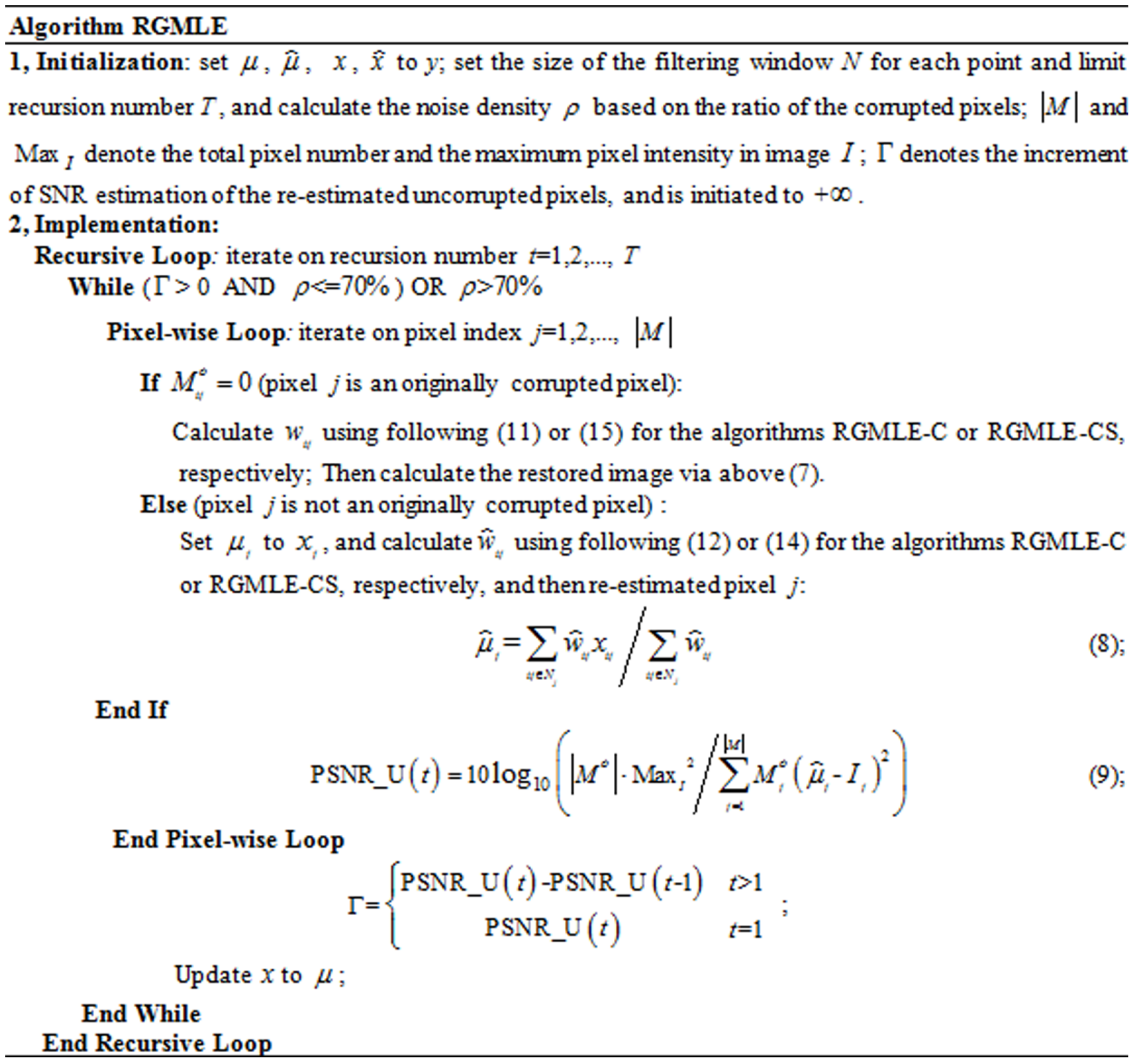
The flowchart of the proposed RGMLE strategy.

The recursive update of image 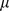 in (7) should be stopped at certain recursion number to avoid the deterioration caused by the successive involvement of those irrelevant uncorrupted pixels. But the original true image is not available to guide the recursion to stop at the point when the optimal restoration (the closest to the original true image) is reached. Here, we use the estimation error of the ground truth uncorrupted pixels to evaluate the restoration deterioration in recursion. After each recursion, we recalculate the intensities of the uncorrupted pixels using the restored image via (7), and analyze their peak signal-to-noise ratio with respect to the original ground truth uncorrupted pixels (PSNR_U, calculated via (9) in the below algorithm outline). The recursion is stopped once the PSNR_U stops to increase, which represents the time when the contribution from corrected pixels starts to be outweighted by the deterioration from the irrelevant uncorrupted pixels. This strategy works well for the cases with noise densities ranging from 10% to 70%. For the cases with very high noise densities (>70%) this strategy does not work well because the re-estimation of the uncorrupted pixels mainly come from irrelevant pixel intensities, and can not reflect the recursion deterioration. In this case a large number of recursion is required to get a stable restoration. An illustration will be given in the below experiment to validate this. We also assume intensity value 255 and 0 for the corrupted pixels in the original image *y*, and let 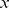 denote the image to be restored. Two binary matrixes 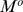 and 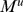 represent the originally uncorrupted pixel mask and the uncorrupted pixel mask in recursion. We should note that the corrupted pixels will receive some estimation and become uncorrupted pixels when they receive some estimation in the processing. So 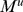 will be updated to an all-ones matrix when all corrupted pixels get estimated as the recursion proceeds. 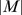, 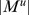 and 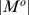 denote the total pixel number in image, and the total non-zero pixel numbers in 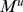 and 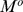, respectively. The flowchart of the RGMLE strategy is given in Fig.2. Here, a large recursion number *T* is set when the noise density is larger than 70%. We judge the PSNR increase for the estimation of the originally uncorrupted pixels by evaluating the error improvement 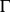. The two algorithms RGMLE-C and RGMLE-CS are detailed as follows:

### Algorithm RGMLE-C

In RGMLE-C algorithm, we consider the certainty measure as whether the pixels are corrupted in the original image *y*. In estimating each 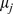 via (7), the variance 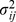 is estimated based on the certainty measure of each observation 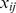, and can be calculated as follows:
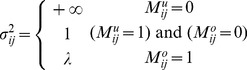
(10)where the pixel 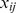 is considered having no certainty if it is originally corrupted and has not yet been corrected (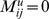), and in this case the variance 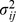 is set to 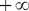. We set 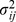 to 1 if the originally corrupted pixel 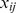 has obtained some certainty via the recursive estimation (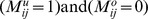). As to those originally uncorrupted pixels (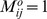), they should be assigned a high certainty, then a low variance is assigned to them with certainty parameter 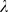 (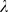
<1). The corresponding weight 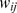 can then be calculated as the reciprocal of variance 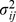:
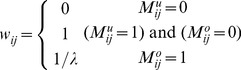
(11)In re-estimating each uncorrupted pixel 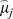 via (8), to accurately reflecting the restoration deterioration in recursion, we exclude the original uncorrupted pixels and only use the restored ones, and the following weight 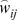
is used:
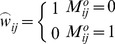
(12)


### Algorithm RGMLE-CS

Within a filter window for one specific point, two pixels belonging to the same structure tend to come from the same class, and should be assigned low variances (or large weights) in estimating each other. We borrow the patch similarity idea in [19], [20], and quantify the variance by the exponential function of the 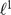 norm distance between the patches centered at the two pixels. Here we use 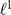 norm for its good edge-preserving property [21], [22]. By introducing this patch similarity information into the variance estimation in the above RGMLE-C algorithm, we calculate each variance 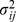:
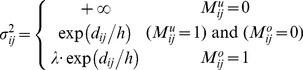
(13)

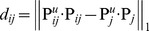
(14)where the variance 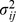 to estimate 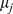 for pixel 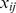 is set to 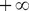 if the corrupted pixel has still not been corrected (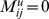). Also if the originally corrupted pixel has obtained some certainty through the correction in recursion (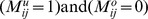), we set the variance to the exponential value of the 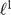 norm of the distance 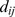 between the uncorrupted pixels in the two 2D patches 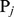 and 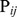 centered at point 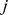 and its neighboring point 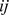. In (14), two binary patch masks 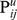 and 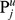 denote the positions of the uncorrupted points and are used to exclude the uncorrupted elements in 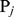 and 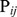 in distance calculation. Practically, parameter *h* in (13) should be set to modulate the attenuation of the exponent function with respect to distance 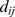. As to the originally uncorrupted pixel (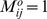), we also lower the value 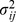 via multiplying it by a certainty parameter 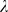 (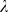
<1). Then, the weight 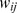 is given as
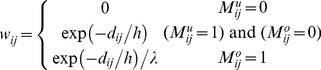
(15)Also, in re-estimating each uncorrupted pixel 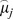, we exclude the original uncorrupted pixels and only use the restored ones. In this case, we use the weight 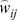
calculated by (16)–(17):



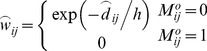
(16)

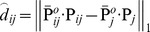
(17)


where, two binary patch masks 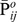 (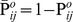) and 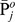 (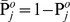) are used to exclude the original uncorrupted pixels in distance calculation.

## Experiments

### A. Experiment configuration

**Figure 3:**
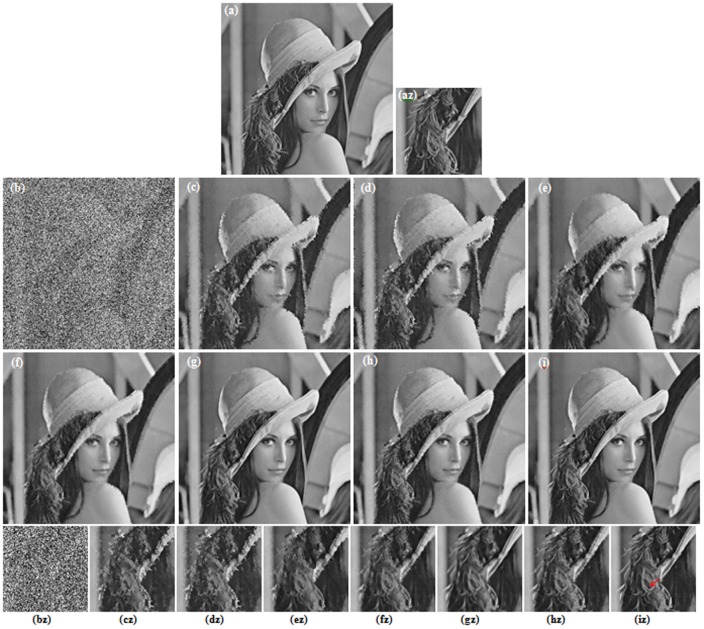
Restoration results of different methods for Lena image (80% salt-and-pepper noise). (a) Original image (b) Corrupted image (6.43 dB, SSIM = 0.0091). (c) AMF (23.44 dB, SSIM = 0.7113) [6]. (d) DBA filter (23.18 dB, SSIM = 0.7112) [7]. (e) MDBUTMF filter (24.51 dB, SSIM = 0.7682) [8]. (f) MDBUTAF filter (27.28 dB, SSIM = 0.8286). (g) SKR method (31.59 dB, SSIM = 0.8793) [23]. (h) RGMLE-C algorithm (29.84 dB, SSIM = 0.8674). (i) RGMLE-CS algorithm (31.54 dB, SSIM = 0.8872). (az)–(iz) denote the zoomed local parts for the corresponding images in (a)–(i).

**Figure 8:**
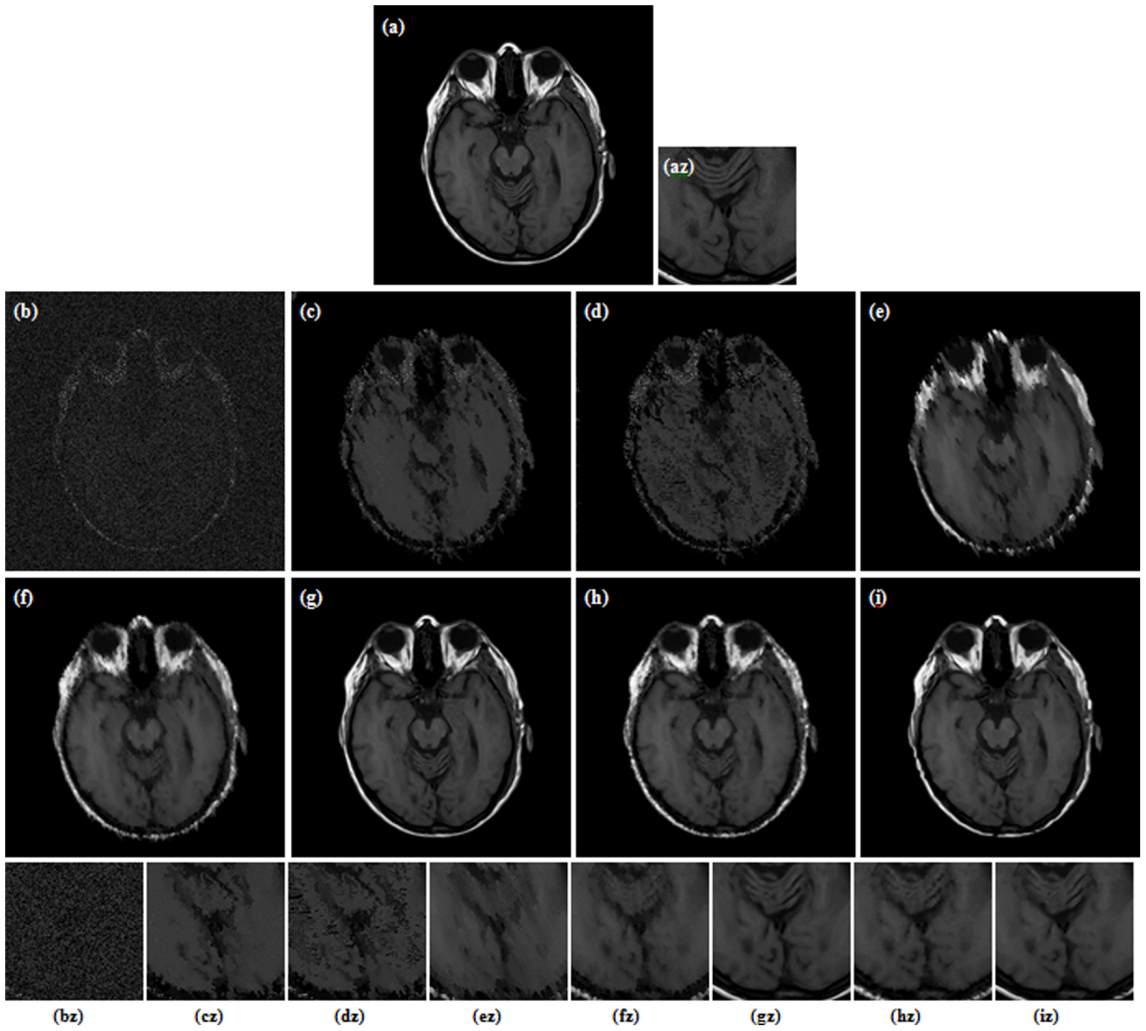
Restoration results for MRI image (90% salt-and-pepper noise). (a) Original image (b) Corrupted image (2.03 dB, SSIM = 0.0096). (c) AMF (6.91 dB, SSIM = 0.6051) [6]. (d) DBA filter (5.91 dB, SSIM = 0.5614) [7]. (e) MDBUTMF filter (9.30 dB, SSIM = 0.6623) [8]. (f) MDBUTAF filter (12.87 dB, SSIM = 0.7532). (g) SKR method (19.40 dB, SSIM = 0.8706) [23]. (h) RGMLE-C algorithm (16.81 dB, SSIM = 0.8426). (i) RGMLE-CS algorithm (18.92 dB, SSIM = 0.8717). (az)–(iz) denote the zoomed local parts for the corresponding images in (a)–(i).

Three 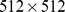
grayscale images (Lena, Pepper and MRI in the below Fig.3–Fig.8) were chosen in the experiments. The Lena and Pepper images were downloaded from http://links.uwaterloo.ca/Repository.html. The intensities of Lena and Peppers images are 8 bit and within the interval [0, 255].The MRI image is in DICOM (Digital Imaging and Communications in Medicine) format and was collected from a head scanning of a 57 years old patient in the first hospital of Nanjing. The intensity range of the MRI image is [0, 1332]. The patient in this manuscript has given the written informed consent of using the MRI image data. A wide range of salt-and-pepper noise with density from 10% to 90% (10% increments) is considered. The same simulation as the analysis in above Section II is used. The AMF, DBA and MDBUTMF methods are implemented based on [6]–[8] for comparison. The MDBUTMF method is actually the standard median filter when any pixel in window is uncorrupted, and switches to averaging filtering otherwise [8]. Replacing the median operator in MDBUTMF by the average operator leads to an average based method and we termed it MDBUTAF. We implement this MDBUTAF method to provide a fair comparison with median and average filtering. We also compare our method with the method based on steering kernel regression (SKR method) in [23], which has demonstrated good performance in denoising, interpolation and super-resolution. The parameters involved in different methods are all set to give stable results with the highest peak signal-to-noise ratio (PSNR, calculated via (18)). We use a 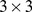 filtering window *N* in the AMF, DBA, MDBUTMF and MDBUTAF methods. For the SKR method, the global smoothing parameter and the kernel size were manually tuned to give the results with the highest PSNR. For the RGMLE-C and RGMLE-CS algorithms, several parameters need to be set, namely the size of filtering window W, the certainty parameter 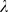 and the recursion limit *T*. Here 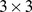 and 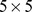 windows are respectively used in the RGMLE-C and RGMLE-CS algorithms. For RGMLE-CS algorithm, the 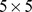 window allows a utilization of more distant pixels via the above similarity-based weighting strategy. Also two additional parameters (the smoothing parameter *h* and the size of patch 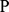) need to be set for the RGMLE-CS method. In RGMLE-CS algorithm, a 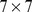 patch 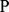 is set to give an effective characterization of local structures. And the smoothing parameter *h* is set to 3.5 for Lena and Peppers images, and to 10 for the MRI image with a larger intensity range. Specially, for RGMLE-CS algorithm, we set 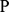 and 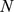 to 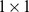 and 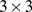 in the first recursion to overcome the lack of structure information in the original corrupted noisy image. We set the certainty parameter 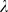 in (11) and (15) to 0.2 to increase the weights of the uncorrupted pixels. The recursion number limit *T* is set to 50 to include a long recursion in restoration using RGMLE-C and RGMLE-CS algorithms.

The pixel-wise operation (7) in RGMLE-C and RGMLE-CS algorithms allows for easy parallelization using GPU technique [24], [25]. Under Compute Unified Device Architecture (CUDA) framework, we set the total number of blocks in grid to the row size of the image, and the total number of threads in each block to the column size of the image. In implementing the RGMLE-C and RGMLE-CS algorithms, all threads in the block-grid structure execute simultaneously to perform all the pixel-wise operations, and only half of the distance 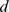 is calculated because of the symmetry property of distance calculation (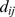 = 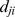). All the images were processed in a PC workstation (Intel Core 2 Quad CPU and 4096 Mb RAM, GPU (NVIDIA GTX465)) with Visual C++ as the developing language (Visual Studio 2008 software; Microsoft).

Restoration performances are quantitatively measured by PSNR and the structural similarity index comparisons (SSIM) proposed in [26]:
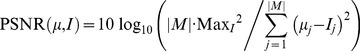
(18)

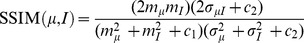
(19)where, 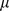 and 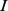 denote the restored image and the original true image. 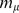 and 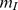 denote the mean intensities of images 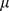
and 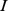, 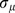
and 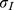
are the standard deviation of images 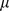 and 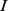, 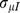 is the covariance of images 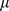 and 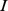, 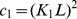
and 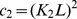 with 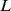 the dynamic range of the pixel values (255 for 8-bit grayscale images), 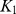 and 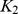 are set to 0.01 and 0.03 as suggested in [25].

### B. Restoration Result

**Figure 4:**
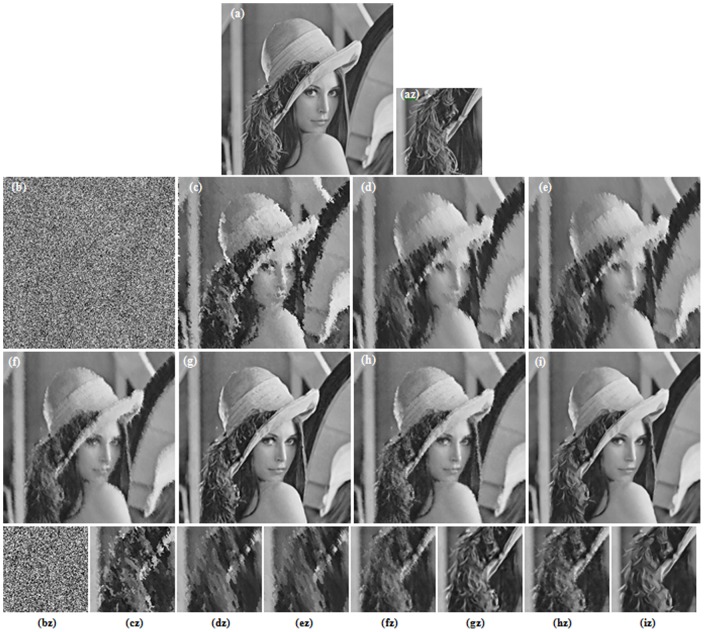
Restoration results of different methods for Lena image (with 90% salt-and-pepper noise). (a) Original image (b) Corrupted image (PSNR = 5.90 dB, SSIM = 0.0052). (c) AMF (17.72 dB, SSIM = 0.5762) [6]. (d) DBA filter (19.52 dB, SSIM = 0.5751) [7]. (e) MDBUTMF filter (20.18 dB, SSIM = 0.6233) [8]. (f) MDBUTAF filter (23.82 dB, SSIM = 0.7373). (g) SKR method (28.52 dB, SSIM = 0.8297) [23]. (h) RGMLE-C algorithm (27.25 dB, SSIM = 0.8084). (i) RGMLE-CS algorithm (28.52 dB, SSIM = 0.8318). (az)–(iz) denote the zoomed local parts for the corresponding images in (a)–(i).

**Figure 5:**
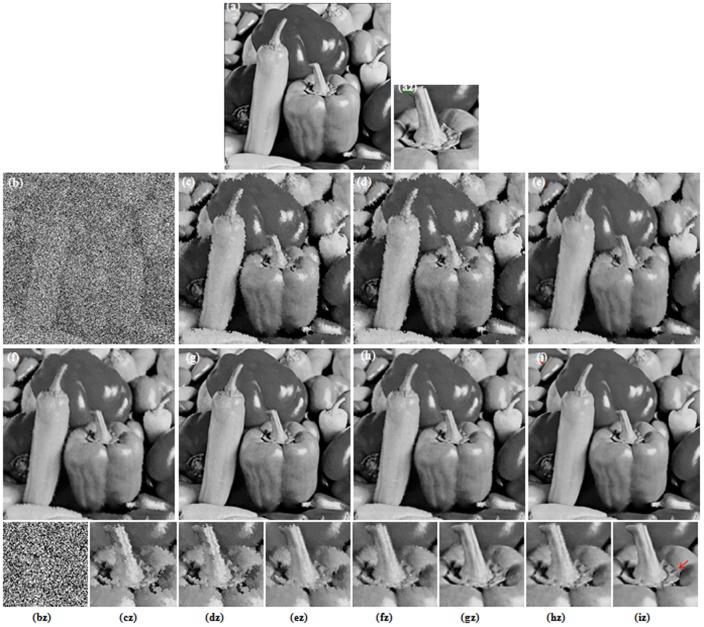
Restoration results of different methods for Peppers image (80% salt-and-pepper noise). (a) Original image (b) Corrupted image (6.26 dB, SSIM = 0.0093). (c) AMF (22.85 dB, SSIM = 0.6581) [6]. (d) DBA filter (22.65 dB, SSIM = 0.6592) [7]. (e) MDBUTMF filter (23.78 dB, SSIM = 0.7174) [8]. (f) MDBUTAF filter (26.72 dB, SSIM = 0.7805). (g) SKR method (31.16 dB, SSIM = 0.8094) [23]. (h) RGMLE-C algorithm (29.97 dB, SSIM = 0.8194). (i) RGMLE-CS algorithm (32.01 dB, SSIM = 0.8381). (az)–(iz) denote the zoomed local parts for the corresponding images in (a)–(i).

**Figure 6:**
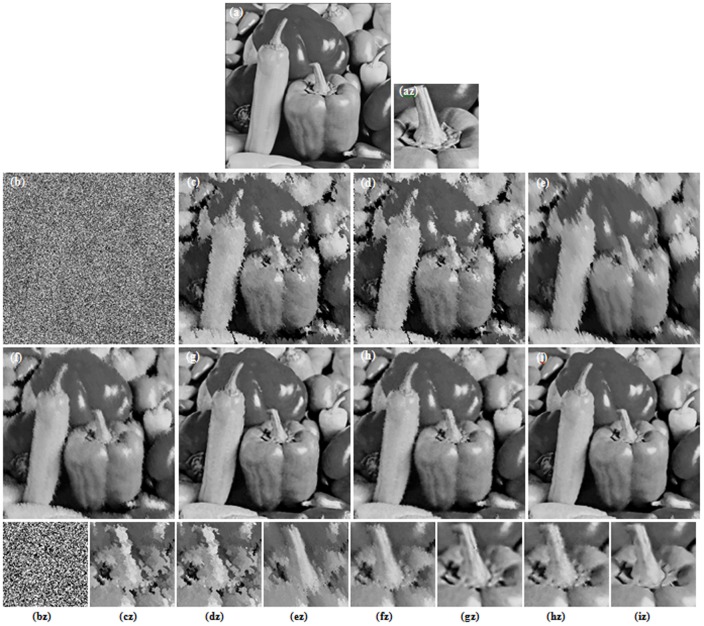
Restoration results of different methods for Peppers image (90% salt-and-pepper noise). (a) Original image (b) Corrupted image (PSNR = 5.84 dB, SSIM = 0.0061). (c) AMF (19.02 dB, SSIM = 0.5205) [6]. (d) DBA filter (18.94 dB, SSIM = 0.5232) [7]. (e) MDBUTMF filter (19.01 dB, SSIM = 0.5657) [8]. (f) MDBUTAF filter (22.92dB, SSIM = 0.6824). (g) SKR method (29.11 dB, SSIM = 0.7765) [23]. (h) RGMLE-C algorithm (27.56 dB, SSIM = 0.7649). (i) RGMLE-CS algorithm (28.83 dB, SSIM = 0.7915). (az)–(iz) denote the zoomed local parts for the corresponding images in (a)–(i).

**Figure 7:**
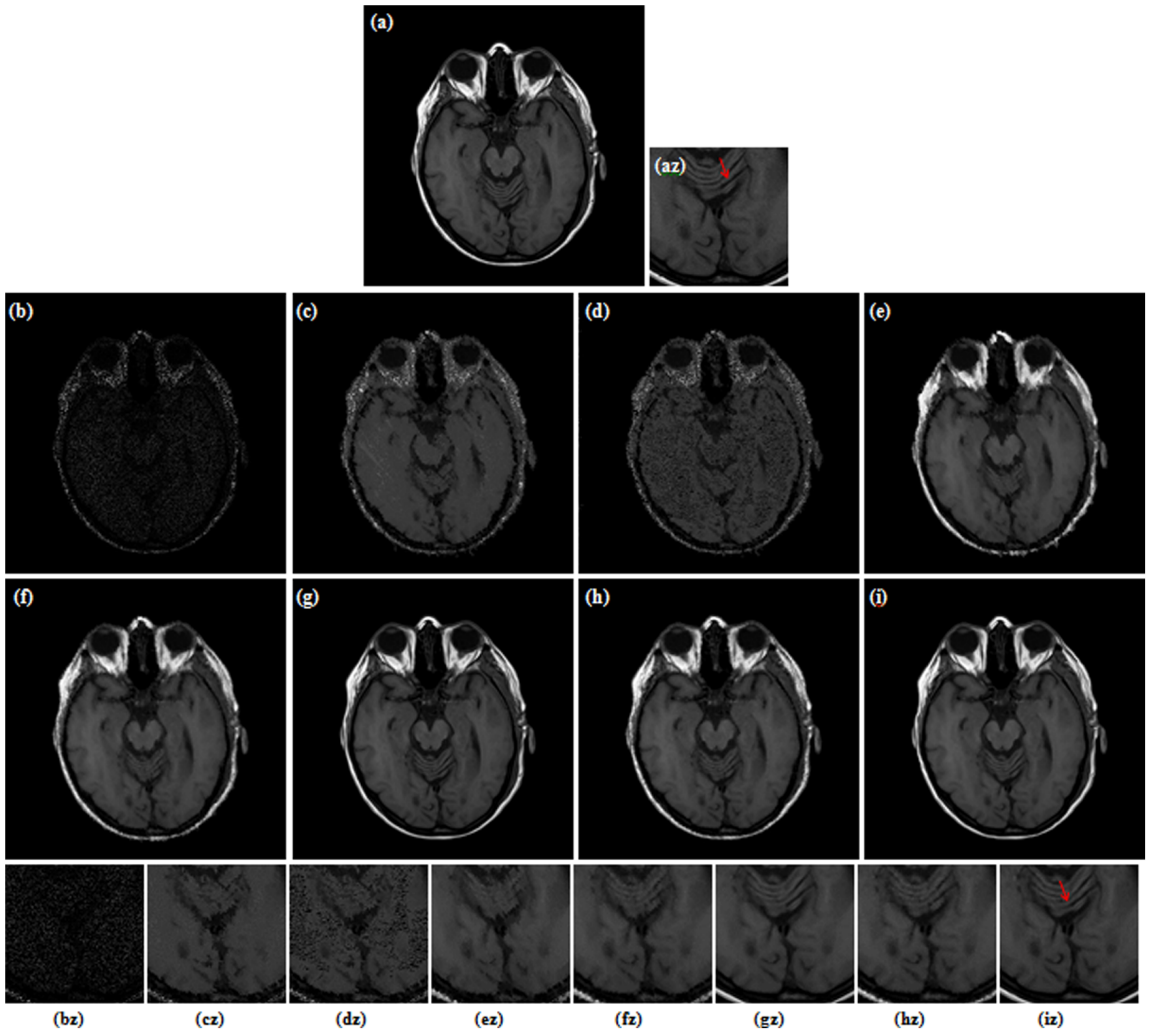
Restoration results for MRI image (80% salt-and-pepper noise). (a) Original image (b) Corrupted image (2.54 dB, SSIM = 0.0195). (c) AMF (8.53 dB, SSIM = 0.6889) [6]. (d) DBA filter (6.26 dB, SSIM = 0.6191) [7]. (e) MDBUTMF filter (13.68 dB, SSIM = 0.7981) [8]. (f) MDBUTAF filter (16.54 dB, SSIM = 0.8524). (g) SKR method (24.22 dB, SSIM = 0.9203) [23]. (h) RGMLE-C algorithm (19.74 dB, SSIM = 0.9012). (i) RGMLE-CS algorithm (23.76 dB, SSIM = 0.9226). (az)–(iz) denote the zoomed local parts for the corresponding images in (a)–(i).

Fig. 3 and Fig. 4 illustrate the restoration results of Lena image with 80% and 90% noise densities for different methods. Likewise, Fig. 5 and Fig. 6 present the results for Pepper image, and Fig. 7 and Fig. 8 present the results for MRI image. The calculated PSNR and SSIM with respect to the original true images are also given in the captions below the figures. The original true images and the corrupted images are illustrated in (a) and (b) for reference. In Fig.3–Fig.8, the denoised images from different methods are given in (c)–(i), and (az)–(iz) denote the zoomed local parts for the corresponding images in (a)–(i).We can see that the traditional median-type filters (AMF, DBA and MDBUTMF methods with results (c)–(e) in Fig.3–Fig.8) can not effectively estimate corrupted points, and tend to introduce many artifacts in restoration. Comparing the images (e) and (f) in Fig.3–Fig.8, we find that under high noise densities the MDBUTAF method works much better than MDBUTMF method (over 2.5dB PSNR enhancement). But we also find the MDBUTMF method with non-discriminative averaging operation can not effectively restore image details and overcome oversmoothing. Comparing the images (f) and (g) in Fig.3–Fig.8, we can see that, by recursively incorporating the certainty measure into estimation, the RGMLE-C algorithm produces notable improvement with more than 2.5dB PSNR enhancement over the MDBUTAF method.

Obviously, the proposed RGMLE-CS algorithm gives good performance in terms of both noise suppression and detail preservation(see images (i) in Fig.3–Fig.8). With patch similarity information used, some tiny features (e. g. the hair structures in Lena image and the brain structures in MRI image) can be successfully restored by the RGMLE-CS algorithm. Among all the methods, the RGMLE-CS algorithm leads to the results with significantly higher PSNR values than other methods except the SKR method. We note that SKR method can give higher PSNR values than the RGMLE-CS algorithm in Lena image (80% noise density), Peppers image (90% noise density) and MRI image (80% and 90% noise density). But in terms of SSIM, which is deemed a more robust quality metric than PSNR, the proposed RGMLE-CS algorithm provides the best results. Compared to the results from SKR method, the processed results from RGMLE-CS algorithm present structures with higher contrast in the case of 80% noise density (see the arrows in images (iz) in Fig.3, Fig.5 and Fig.7), and perceptively comparable results under 90% noise density.

**Figure 9:**
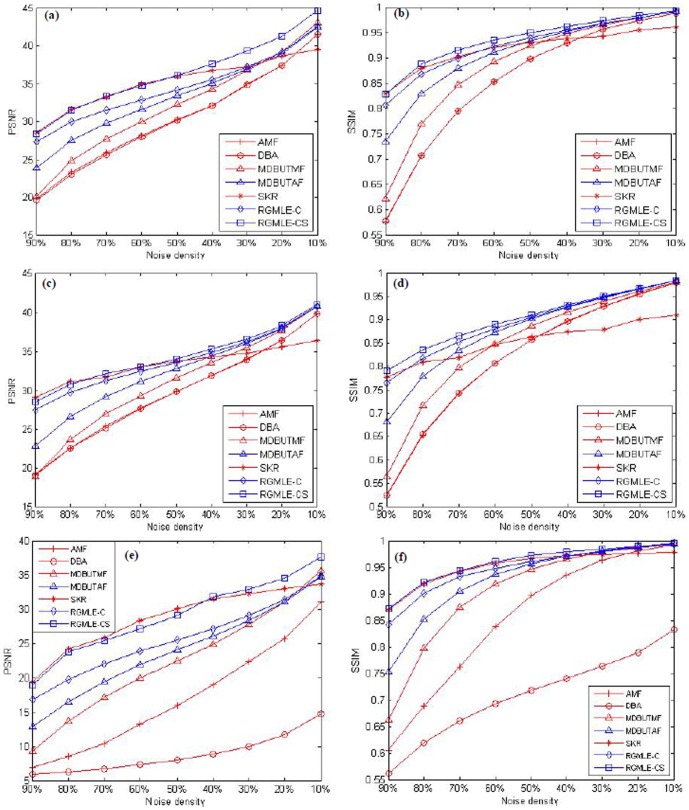
PSNR and SSIM values for various noise densities for different algorithms. (a) and (b): the PSNR and SSIM for Lena image; (c) and (d): the PSNR and SSIM for Pepper image; (e) and (f): the PSNR and SSIM for MRI image.

In Fig. 9, we summarize the PSNR and SSIM values of the restored images for noise densities ranging from 10% to 90%. We can observe in the plots that the obtained PSNR and SSIM values decrease as noise level rises for all the methods, and our proposed RGMLE-C and RGMLE-CS algorithms obtain significantly higher PSNR and SSIM than the other methods except the SKR method in almost all noise densities. This advantage becomes more prominent as noise density increases. The SKR method can sometimes provide higher PSNR values than RGMLE-CS algorithm under high noise densities (80% and 90%), but has poor performance in the cases of low noise densities. We can see the proposed RGMLE-CS algorithm can give the best results in terms of SSIM for all the noise densities.

### C. Validation of Recursion Stopping

**Figure 10:**
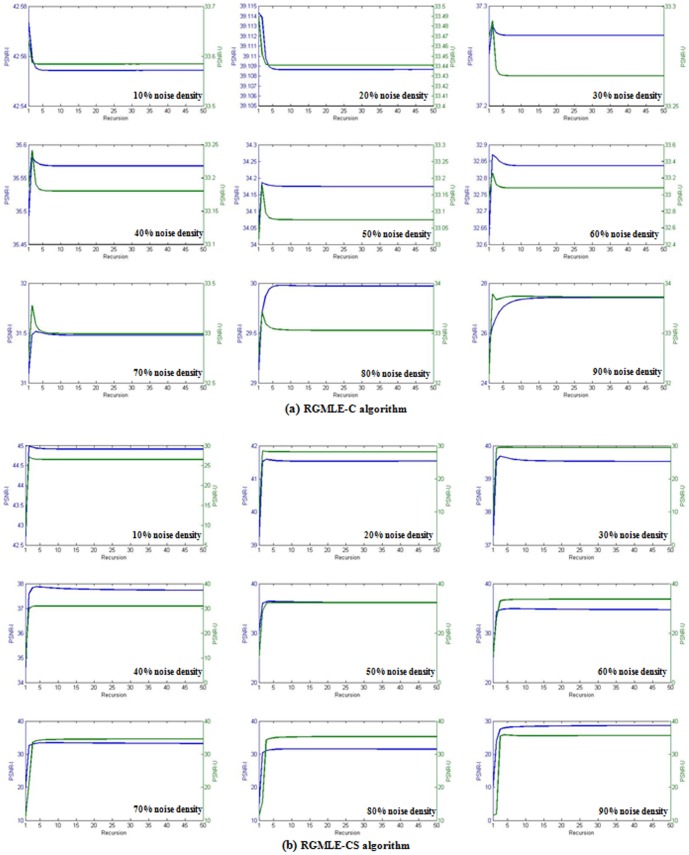
For Lena image test, the PSNR of the whole restored image (PSNR-I) and the re-estimated uncorrupted pixels (PSNR-U) as the recursion proceeds for noise densities from 10% to 90%. (a) the result of RGMLE-C algorithm; (b) the result of RGMLE-CS algorithm.

Fig. 10 plots the PSNR (via (18)) with respect to the recursion numbers (*T* = 50) for the whole restored image (denoted by PSNR_I, blue lines) and the PSNR_U (via above (9)) of the re-estimated uncorrupted pixels (green lines) under different noise densities. Lena image with the same parameter setting as above is used in this validation. Fig. 10 shows that the PSNR-I decreases when the recursion proceeds across one specific point, which shows the deterioration will appear in recursion for the proposed RGMLE-C and RGMLE-CS methods. In Fig. 10, we can note a good match between the highest obtained PSNR of the restored images and that of the re-estimated uncorrupted pixels, but such match degree tends to obviate as the noise density increases to over 70%. The reason of the match deviation for noise density higher than 70% is that under very high noise densities the re-estimation of the uncorrupted pixels mainly relies on irrelevant pixel intensities, which can not accurately reflect the overall deterioration in restoration. This observation validates the above proposed stopping strategy for the RGMLE algorithm.

### D. Computation Complexity

Table 1 lists the CPU time costs in implementing all the methods in the above experiments with different noise densities. The CPU time costs for implementing both the parallelized RGMLE methods (parallelized RGMLE-C and RGMLE-CS) and the original serial versions (serial RGMLE-C and RGMLE-CS) are included. The AMF and SKR methods require much more intensive computation than other median-type filters because the AMF method recursively enlarges the filter window to obtain an uncorrupted median and the SKR method requires a computationally costly operation of matrix inversion. Table 1 also shows that the proposed RGMLE-C and RGMLE-CS methods require more computation than other methods, and about 150 times acceleration is achieved after GPU based parallelization. We can also notice that the required computation cost of RGMLE-C and RGMLE-CS methods increases as the noise density increases because more recursions need to be run based on the recursion stopping rule.

**Table 1:** Computation Time (In CPU Seconds) For Different Methods With Different Noise Densities.

Images and noise levels	AMF	DBA	MDBUTMF	MDBUTAF	SKR	Serial RGMLE-C	Serial RGMLE-CS	Parallelized RGMLE-C	Parallelized RGMLE-CS
**Lena (10% noise density)**	3.35	3.04	1.99	1.99	35.58	**13.63**	**107.73**	**0.14**	**0.85**
**Lena (30% noise density)**	4.61	3.16	3.26	3.44	94.93	**21.69**	**197.45**	**0.23**	**1.27**
**Lena (50% noise density)**	8.86	3.13	4.39	4.61	106.53	**22.72**	**400.73**	**0.24**	**2.65**
**Lena (70% noise density)**	27.22	3.18	5.56	5.95	86.86	**23.61**	**998.64**	**0.26**	**11.99**
**Lena (90% noise density)**	85.53	3.22	7.25	7.20	69.76	**78.53**	**1109.16**	**0.83**	**12.88**
**Peppers (10% noise density)**	30.07	3.05	2.09	1.96	36.41	**13.75**	**104.67**	**0.15**	**0.85**
**Peppers (30% noise density)**	48.54	3.12	3.18	3.52	86.89	**21.69**	**203.78**	**0.23**	**1.35**
**Peppers (50% noise density)**	48.23	3.19	4.52	4.69	103.01	**22.62**	**225.26**	**0.27**	**1.46**
**Peppers (70% noise density)**	53.25	3.19	5.61	5.99	87.72	**23.50**	**233.47**	**0.29**	**1.64**
**Peppers (90% noise density)**	94.87	3.24	7.26	7.28	72.23	**80.85**	**1218.09**	**0.91**	**14.13**
**MRI (10% noise density)**	324.60	3.05	1.99	1.96	82.10	**13.68**	**104.89**	**0.15**	**0.89**
**MRI (30% noise density)**	377.83	3.22	3.24	3.44	74.05	**22.07**	**198.37**	**0.24**	**1.32**
**MRI (50% noise density)**	307.07	3.22	4.50	4.82	88.65	**23.18**	**215.40**	**0.25**	**1.41**
**MRI (70% noise density)**	222.87	3.21	5.70	6.00	82.70	**23.61**	**238.47**	**0.28**	**1.66**
**MRI (90% noise density)**	167.03	3.33	7.39	7.39	71.93	**79.44**	**1201.26**	**0.89**	**13.52**

## Conclusions

This paper proposed a Recursive Gaussian Maximum Likelihood Estimation (RGMLE) for 2-D impulse noise removal. Assuming Gaussian distribution for neighboring intensities, the proposed RGMLE approach solves the maximum likelihood estimation (MLE) of corrupted points through recursive variance-weighted averaging. Simulation in Section II reveals that Gaussian distribution assumption is theoretically preferable to Laplacian distribution assumption in estimating corrupted points under high noise densities. In validation we use salt-and-pepper type impulse noise with pre-known intensities. Two noise removal algorithms RGMLE-C and RGMLE-CS are respectively derived by using certainty based or certainty&similarity based variance estimations. An effective stopping strategy for recursion is also developed based on the estimation error of uncorrupted pixels using restored image information. Extensive experiments have been performed to show the RGMLE-C and RGMLE-CS algorithm can obtain significantly better results than the comparative median type filters. Among all the methods, the RGMLE-CS algorithm achieves good restoration performance through the incorporation of patch similarity information. The application of the proposed RGMLE methods can be easily extended to suppress general impulse noise with random values when combined with the detection methods proposed in [3], [4], [11], [27]. An extension to Gaussian noise suppression would also be performed by considering a spatially varying noise corruption degree for each pixel.

With GPU parallelization, the RGMLE-CS algorithm can be efficiently applied and should be suggested for impulse noise suppression. A constant smoothing parameter *h* is currently used in the RGMLE-CS algorithm, and we can improve the algorithm by optimizing the estimation of parameter *h* [28], [29]. The denoising performance of the RGMLE-CS algorithm can also be further enhanced via the combination with multiscale or compressed sensing techniques [30], [31]. Current recursion stopping method is based on the knowledge of estimation error of uncorrupted pixels, and from above we know that this strategy does not apply to the case of very high noise density. More effective method is to be studied to control recursion to obtain the exactly optimal restoration for different noise levels. Computation efficiency still holds a future issue to be considered. Though significant acceleration has been obtained by GPU parallelization technique, further computation optimization is still required to realize real-time processing of the RGMLE-CS algorithm.
